# Emergency Department Peer Support Program and Patient Outcomes After Opioid Overdose

**DOI:** 10.1001/jamanetworkopen.2024.3614

**Published:** 2024-03-25

**Authors:** Peter Treitler, Stephen Crystal, Joel Cantor, Sujoy Chakravarty, Anna Kline, Cory Morton, Kristen Gilmore Powell, Suzanne Borys, Nina A. Cooperman

**Affiliations:** 1 Institute for Health, Health Care Policy and Aging Research, Rutgers University, New Brunswick, New Jersey; 2 Boston University School of Social Work, Boston, Massachusetts; 3 School of Social Work, Rutgers University, New Brunswick, New Jersey; 4 School of Public Health, Rutgers University, Piscataway, New Jersey; 5 Department of Health Sciences, Rutgers University, Camden, New Jersey; 6 Department of Psychiatry, Rutgers Robert Wood Johnson Medical School, Piscataway, New Jersey; 7 Center for Prevention Science, Rutgers University, New Brunswick, New Jersey; 8 Northeast and Caribbean Prevention Technology Transfer Center, Rutgers University, New Brunswick, New Jersey; 9 Division of Mental Health and Addiction Services, New Jersey Department of Human Services, Trenton

## Abstract

**Importance:**

Patients treated in emergency departments (EDs) for opioid overdose often need drug treatment yet are rarely linked to services after discharge. Emergency department–based peer support is a promising approach for promoting treatment linkage, but evidence of its effectiveness is lacking.

**Objective:**

To examine the association of the Opioid Overdose Recovery Program (OORP), an ED peer recovery support service, with postdischarge addiction treatment initiation, repeat overdose, and acute care utilization.

**Design, Setting, and Participants:**

This intention-to-treat retrospective cohort study used 2014 to 2020 New Jersey Medicaid data for Medicaid enrollees aged 18 to 64 years who were treated for nonfatal opioid overdose from January 2015 to June 2020 at 70 New Jersey acute care hospitals. Data were analyzed from August 2022 to November 2023.

**Exposure:**

Hospital OORP implementation.

**Main Outcomes and Measures:**

The primary outcome was medication for opioid use disorder (MOUD) initiation within 60 days of discharge. Secondary outcomes included psychosocial treatment initiation, medically treated drug overdoses, and all-cause acute care visits after discharge. An event study design was used to compare 180-day outcomes between patients treated in OORP hospitals and those treated in non-OORP hospitals. Analyses adjusted for patient demographics, comorbidities, and prior service use and for community-level sociodemographics and drug treatment access.

**Results:**

A total of 12 046 individuals were included in the study (62.0% male). Preimplementation outcome trends were similar for patients treated in OORP and non-OORP hospitals. Implementation of the OORP was associated with an increase of 0.034 (95% CI, 0.004-0.064) in the probability of 60-day MOUD initiation in the half-year after implementation, representing a 45% increase above the preimplementation mean probability of 0.075 (95% CI, 0.066-0.084). Program implementation was associated with fewer repeat medically treated overdoses 4 half-years (−0.086; 95% CI, −0.154 to −0.018) and 5 half-years (−0.106; 95% CI, −0.184 to −0.028) after implementation. Results differed slightly depending on the reference period used, and hospital-specific models showed substantial heterogeneity in program outcomes across facilities.

**Conclusions and Relevance:**

In this cohort study of patients treated for opioid overdose, OORP implementation was associated with an increase in MOUD initiation and a decrease in repeat medically treated overdoses. The large variation in outcomes across hospitals suggests that treatment effects were heterogeneous and may depend on factors such as implementation success, program embeddedness, and availability of other hospital- and community-based OUD services.

## Key Points

**Question:**

Is implementation of an emergency department (ED)–based peer recovery support program for opioid overdose associated with improvements in initiation of medication for opioid use disorder (MOUD)?

**Findings:**

In this cohort study of 12 046 patients treated for nonfatal opioid overdose, those treated in EDs that implemented peer support were significantly more likely to initiate MOUD than patients treated in comparison EDs. The outcome varied across EDs and by time since peer support implementation.

**Meaning:**

The findings suggest that ED-based peer recovery support is associated with increased postdischarge MOUD receipt, but outcomes likely depend on additional factors such as program characteristics and availability of other substance use disorder services.

## Teaser

This cohort study investigates the association of an emergency department peer recovery support service with postdischarge addiction treatment initiation among patients admitted for nonfatal opioid overdose in New Jersey.

## Introduction

Opioid overdose deaths have continued to increase and exceeded 80 000 in 2021.^1^ Nonfatal opioid overdoses are more common than and often precede fatal overdoses.^2,3,4,5^ Many individuals surviving an opioid overdose are treated in emergency departments (EDs), critical settings in which to intervene and engage patients in services at a time when they may be more receptive to help.^6,7^ However, in many cases, patients are discharged without intervention to prevent repeat overdose or link them to treatment. Only 10% to 30% of individuals engage in treatment in the months following a medically treated opioid overdose,^5,8,9,10^ reflecting inadequate intervention in EDs.

Emergency department–based interventions for patients experiencing opioid overdose have been rapidly developed and implemented.^11,12,13^ One approach is to have peers, people who share life experiences with those they serve, deliver interventions in the ED. This role for peers, also referred to as recovery coaches, recovery specialists, or peer mentors, grew out of the idea that lived experience provides individuals with unique insights and the capacity to build rapport with and support others with similar experiences.^14,15,16^ Peers working in dedicated roles are also well positioned to assist ED patients with substance use disorders (SUDs) compared with medical staff with competing priorities constraining their time.^17,18^

Emergency department–based peer recovery support services (PRSSs) are a promising approach for promoting treatment linkage, but evidence regarding their effectiveness is limited. Studies in other settings suggest that PRSSs are associated with reduced alcohol and drug use, improved treatment engagement, and lower likelihood of rehospitalization.^19,20,21,22,23,24^ A PRSS may be an important strategy for addressing opioid use disorder (OUD) in the ED,^25,26^ and pilot studies suggest that PRSSs may promote postdischarge SUD treatment enrollment^27^ and reduce repeat overdose.^28^

Emergency department–based PRSSs are expanding rapidly despite limited evidence. To guide implementation, additional research is needed through both clinical trials and observational studies in usual care settings that use diverse data sources and methods to help assess program effectiveness. To that aim, this study used state Medicaid data and an event study design to examine whether implementation of a statewide ED-based PRSS program was associated with an increase in postdischarge SUD treatment initiation and reductions in repeat medically treated overdoses and all-cause acute care visits.

## Methods

This retrospective cohort study was approved by the Rutgers institutional review board, with the informed consent requirement waived because data were deidentified and originally collected for nonresearch purposes. This study followed the Strengthening the Reporting of Observational Studies in Epidemiology (STROBE) reporting guideline for cohort studies.

### Setting and Intervention

In 2015, the New Jersey Department of Human Services implemented the Opioid Overdose Recovery Program (OORP), a PRSS program that links ED patients to medication for opioid use disorder (MOUD) and other treatment and recovery support services following a nonfatal overdose.^29^ The OORP services are primarily delivered by peers (ie, individuals with SUD lived experience), who conduct an initial bedside intervention in the ED immediately following an opioid overdose. For the next 8 weeks, peers provide nonclinical recovery support, with additional support from patient navigators (ie, case managers) to facilitate service linkages. The OORP programs are currently administered by 14 organizations across New Jersey’s 21 counties, with each organization covering between 1 and 13 hospitals. Core elements of the implementation model (eg, staffing, required activities) were laid out by the funding agency,^30^ but implementation models were adapted across programs and EDs depending on resources and structure.^29,31^ For example, some OORP programs embedded peers within EDs, while others dispatched them to the ED as needed.^31^

### Data and Study Cohort

**Figure 1.**
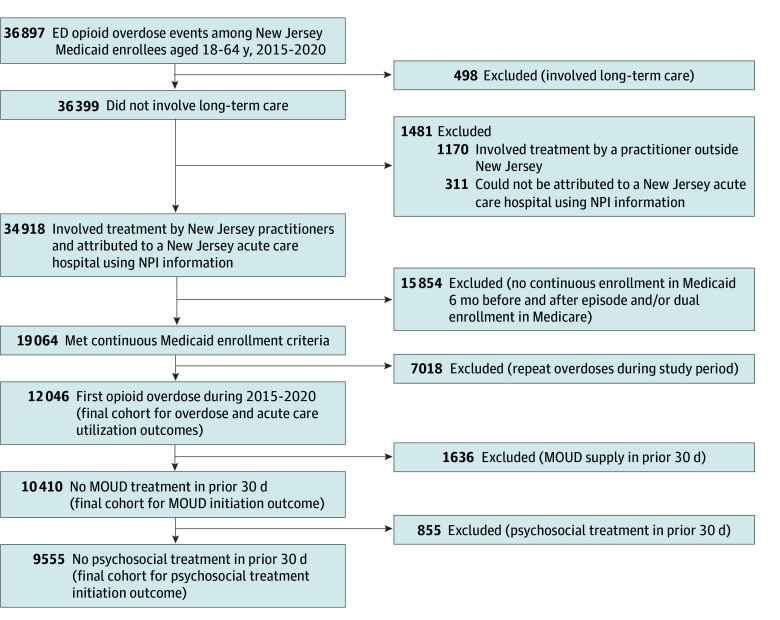
Cohort Flow Diagram ED indicates emergency department; MOUD, medication for opioid use disorder; NPI, National Provider Identifier.

This study used deidentified 2014 to 2020 New Jersey Medicaid claims data. The study cohort included Medicaid beneficiaries aged 18 to 64 years who had an index opioid overdose ED visit from January 2015 to June 2020, identified using diagnosis codes (eTable 1 in Supplement 1). Race and ethnicity, ascertained from a field in the Medicaid claims dataset (eMethods in Supplement 1), were included in the analysis to account for known disparities in treatment access and OUD-related outcomes. Categories were Black, Hispanic, White, and other or unknown (American Indian, Asian, ambiguous categories, and those classified as other or unknown in the Medicaid data). Data from 2014 were included for the 180-day lookback period for overdoses in the first half of 2015. Emergency department and inpatient visits separated by fewer than 2 days were combined into 1 episode. Patients were attributed to 1 of 70 New Jersey acute care hospitals using practitioner and facility information (eMethods in Supplement 1). Patients were included only for their first overdose during the study period and had to be continuously enrolled in Medicaid, without dual Medicare enrollment, for 180 days before and 180 days after the index visit to capture baseline characteristics and outcomes. Patients with SUD treatment in the prior 30 days were excluded from treatment initiation outcomes analyses, as shown in the cohort flow diagram (Figure 1).

### Outcomes and Measures

The primary study outcome was MOUD initiation within 60 days of discharge from the index overdose visit, aligning with the OORP program duration. Secondary outcomes included psychosocial treatment initiation within 60 days,^32^ number of medically treated drug overdoses within 180 days, and number of all-cause acute care visits within 180 days. Outcomes were identified using National Drug Codes and *Current Procedural Terminology* and Healthcare Common Procedure Coding System codes (eTable 2 in Supplement 1).

The study exposure was hospital implementation of the OORP program. Data were aggregated to half-year (6-month) periods, and for each half-year, hospitals were categorized as implementers or nonimplementers. Analyses were adjusted for patient-level (eg, demographics, comorbidities, diagnoses, and prior health service use) and community-level (eg, sociodemographics, drug treatment access) characteristics to account for hospital differences in patient case mix and community factors that may be associated with outcomes (eTable 1 and eMethods in Supplement 1).

### Statistical Analysis

We first calculated descriptive statistics for the study cohort. We then calculated unadjusted means in outcomes for intervention and comparison groups before and after OORP implementation (eMethods in Supplement 1). Next, we used an event study design to compare patient outcomes in hospitals that implemented the OORP with outcomes of patients in non-OORP hospitals. The design took advantage of the phased implementation of the program in 53 hospitals over 7 waves occurring during a 5-year period, allowing hospitals that later implemented the program to contribute comparison group observations before implementation.

Because of the bias associated with event study designs that use 2-way fixed-effects models for staggered implementation,^33^ we used the stacked regression estimator.^34,35^ We organized data into subsets to create separate datasets for each of the 7 implementation waves occurring in distinct half-year periods. Each dataset included overdoses treated in hospitals that implemented the OORP during the given wave (intervention group) and overdoses treated in hospitals that had never or not yet implemented the OORP as of that wave (comparison group). Observations, comprising overdoses in each dataset, contained time indicators of the half-year of overdose relative to program implementation. Period −1 represented the half-year before program implementation; period 0, the half-year of implementation; period 1, the half-year after implementation; and so on. Half-years −9 to −6 and 6 to 8 were aggregated for model parsimony. Because time indicators are relative to the half-year of program implementation, they represent estimated treatment effects at defined periods before and after implementation regardless of the calendar time when the program was implemented in each wave. A dataset-specific identifier was added to each dataset, and datasets were stacked (ie, appended) into a single dataset. We estimated an event study model using this full dataset with dataset-specific hospital and time fixed effects (to account for repeated observations in the stacked dataset) as well as individual- and hospital-level controls.^34^ Outcomes were modeled in a linear regression framework, with sensitivity analyses using binomial and quasi-Poisson regression for dichotomous and count outcomes, respectively. To avoid perfect collinearity, we omitted the half-year of program implementation (period 0), and treatment effect estimates are relative to this omitted period. Standard errors were clustered on hospital and patient zip code. The analysis followed an intention-to-treat design, wherein patients were in the intervention group if they were treated in an implementation hospital.

To assess variability in program outcomes across hospitals, we performed hospital-specific 2 × 2 difference-in-differences (DID) analyses for the 60-day MOUD and 180-day overdose outcomes. Analyses were limited to 37 hospitals with at least 30 observations during the study period. For each model, observations of treatment for overdose in the intervention hospital comprised the treatment group, and observations in all other hospitals that had never or not yet implemented the OORP comprised the comparison group. Because of the relatively small sample size within hospitals, we aggregated observations into preimplementation and postimplementation periods and conducted 2 × 2 DID analyses. Models used linear regression with SEs clustered on hospital and patient zip code.

We performed several sensitivity analyses to assess robustness of findings to alternate cohort specifications, comparison groups, reference periods, and event study models. Data were analyzed from August 2022 to November 2023 using SAS Enterprise Guide, version 8.3 (SAS Institute Inc), and StataMP, version 17 (StataCorp LLC). Analyses used a 2-tailed significance threshold of *P* < .05. Additional details regarding study measures, the statistical approach, and sensitivity analyses are described in the eMethods in Supplement 1.

## Results

### Sample Characteristics

Of 12 046 eligible patients treated for opioid overdose, 55.1% were aged 25 to 44 years, 25.2% were Black, 9.3% were Hispanic, 57.7% were White, 7.8% were other or unknown race and ethnicity, 38.0% were female, and 62.0% were male (Table). Most had a diagnosis of OUD (51.5%), other SUD (57.5%), and/or a psychiatric illness (59.3%). In the 180 days before the overdose, 24.6% of patients received an MOUD and 25.1% received psychosocial treatment. A total of 69.5% of index overdoses involved heroin or synthetic opioids, and 10.6% involved other substances in addition to opioids. Patients lived within 8 km of a mean (SD) of 30.0 (30.5) buprenorphine prescribers, 10.4 (10.2) specialty SUD treatment facilities, and 1.6 (2.0) methadone programs. Differences in characteristics of the intervention and comparison groups are shown in the Table.

**Table.** Cohort Description^a^

Characteristic	Participants, No. (%)			*P* value
	Total (N = 12 046)	Intervention group (n = 5868)	Comparison group (n = 6178)	
Age, y				
18-24	1053 (8.7)	446 (7.6)	607 (9.8)	<.001
25-34	4005 (33.2)	1943 (33.1)	2062 (33.4)	
35-44	2643 (21.9)	1373 (23.4)	1270 (20.6)	
45-54	2721 (22.6)	1338 (22.8)	1383 (22.4)	
55-64	1624 (13.5)	768 (13.1)	856 (13.9)	
Race and ethnicity				
Black	3035 (25.2)	1404 (23.9)	1631 (26.4)	<.001
Hispanic	1121 (9.3)	618 (10.5)	503 (8.1)	
White	6947 (57.7)	3441 (58.6)	3506 (56.7)	
Other or unknown^b^	943 (7.8)	405 (6.9)	538 (8.7)	
Sex				
Female	4577 (38.0)	2107 (35.9)	2470 (40.0)	<.001
Male	7469 (62.0)	3761 (64.1)	3708 (60.0)	
Comorbidities				
OUD	6201 (51.5)	3206 (54.6)	2995 (48.5)	<.001
Other SUD	6927 (57.5)	3367 (57.4)	3560 (57.6)	.79
Any psychiatric diagnosis	7143 (59.3)	3482 (59.3)	3661 (59.3)	.93
Chronic pain	6544 (54.3)	3114 (53.1)	3430 (55.5)	.007
Hepatitis C	1948 (16.2)	950 (16.2)	998 (16.2)	.96
HIV infection	392 (3.3)	166 (2.8)	226 (3.7)	.01
CCW conditions, No.				
0	4431 (36.8)	2248 (38.3)	2183 (35.3)	<.001
1-2	4795 (39.8)	2320 (39.5)	2475 (40.1)	
≥3	2820 (23.4)	1300 (22.2)	1520 (24.6)	
Prior 180-d health service use				
Inpatient stay	2993 (24.8)	1416 (24.1)	1577 (25.5)	.08
ED visit	7684 (63.8)	3722 (63.4)	3962 (64.1)	.42
MOUD receipt	2958 (24.6)	1590 (27.1)	1368 (22.1)	<.001
Psychosocial treatment	3024 (25.1)	1610 (27.4)	1414 (22.9)	<.001
Nonopioid overdose	320 (2.7)	146 (2.5)	174 (2.8)	.26
Index event characteristics				
Opioid overdose type				
Heroin or synthetic opioids	8375 (69.5)	4184 (71.3)	4191 (67.8)	<.001
Other opioids only	3671 (30.5)	1684 (28.7)	1987 (32.2)	
Nonopioid overdose	1279 (10.6)	542 (9.2)	737 (11.9)	<.001
Inpatient stay	2690 (22.3)	1160 (19.8)	1530 (24.8)	<.001
Inpatient detox stay	700 (5.8)	271 (4.6)	429 (6.9)	<.001
Inpatient psychiatric stay	75 (0.6)	42 (0.7)	33 (0.5)	.21
Community characteristics				
SDI percentile, mean (SD)^c^	79.2 (91.4)	77.6 (90.7)	80.8 (92.1)	.06
BUP prescribers within 8 km, mean (SD), No.	30.0 (30.5)	32.7 (33.1)	27.4 (27.5)	<.001
SUD treatment facilities within 8 km, mean (SD), No.	10.4 (10.2)	9.7 (9.1)	11.1 (11.0)	<.001
OTPs within 8 km, mean (SD), No.	1.6 (2.0)	1.5 (1.8)	1.7 (2.1)	<.001

Abbreviations: BUP, buprenorphine; CCW, Chronic Conditions Data Warehouse; ED, emergency department; MOUD, medication for opioid use disorder; OTP, opioid treatment program; OUD, opioid use disorder; SDI, Social Deprivation Index; SUD, substance use disorder.

^a^ Reflects the cohort for overdose and all-cause acute care visit outcomes.

^b^ Other or unknown race and ethnicity includes American Indian, Asian, ambiguous categories, and those classified as other or unknown in the Medicaid data.

^c^ Mean percentiles for the communities represented by individuals in the treatment and comparison groups. Communities with a higher SDI percentile are considered more deprived.

### Association of Program Implementation With Outcomes

**Figure 2.**
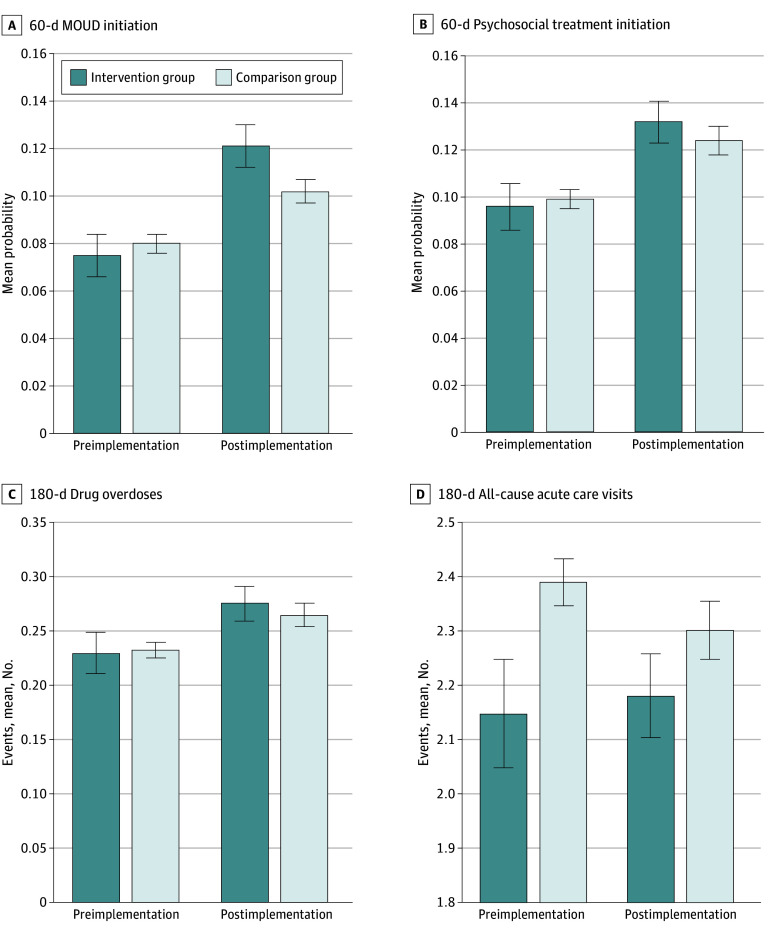
Unadjusted Patient Outcomes Before and After Opioid Overdose Recovery Program Implementation for Intervention and Comparison Groups Error bars represent 95% CIs. MOUD indicates medication for opioid use disorder.

Figure 2 descriptively compares unadjusted means in patient outcomes before and after OORP implementation for the intervention and comparison groups. For all outcomes except 180-day acute care visits, baseline rates and trends before and after OORP implementation were similar. The mean number of acute care visits increased slightly from 2.15 (95% CI, 2.05-2.25) before implementation to 2.18 (95% CI, 2.10-2.26) after implementation in the intervention group while decreasing in the comparison group from 2.39 (95% CI, 2.35-2.43) to 2.30 (95% CI, 2.25-2.36). In the intervention vs the comparison group, mean probability of 60-day MOUD initiation was lower before implementation (0.075 [95% CI, 0.066-0.084] vs 0.080 [95% CI, 0.076-0.084]) and higher after implementation (0.121 [95% CI, 0.112-0.130] vs 0.102 [95% CI, 0.097-0.107]).

**Figure 3.**
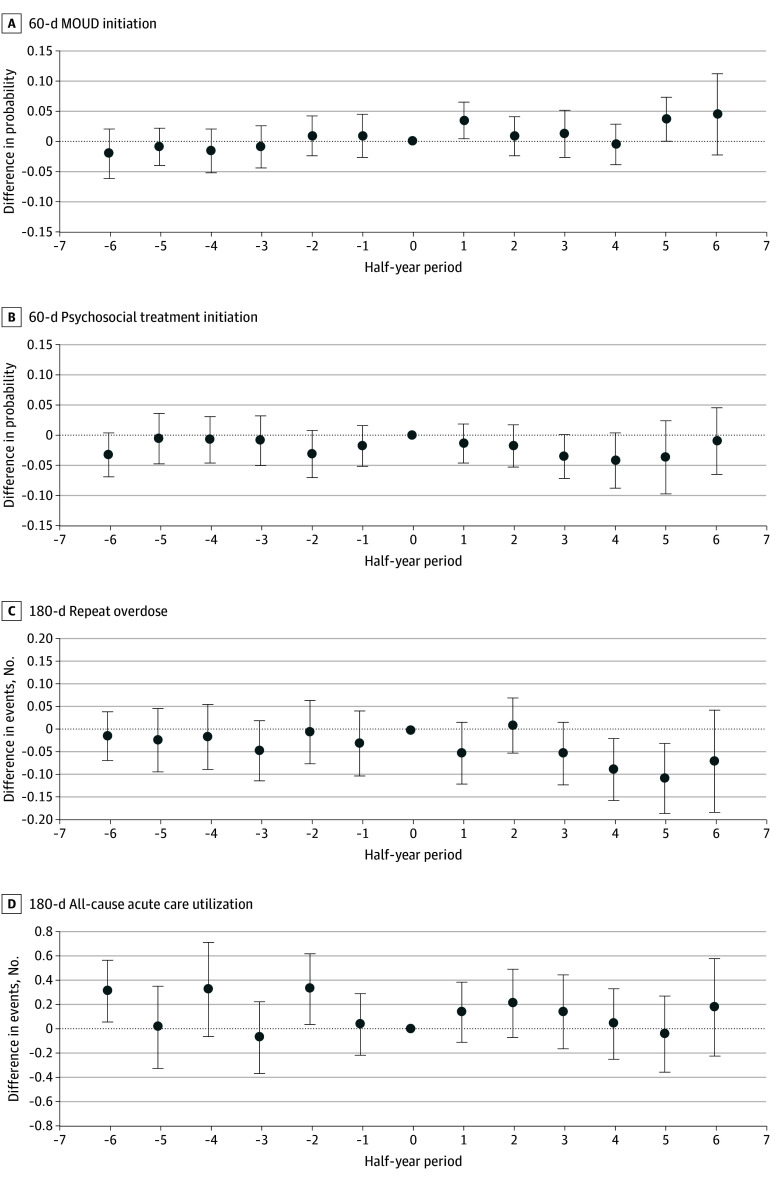
Event Study Plots Showing Associations of Opioid Overdose Recovery Program (OORP) Implementation With Outcomes Half-years are relative to OORP implementation, which is represented as half-year 0. Estimates represent the difference in outcomes relative to the half-year of OORP implementation (time 0) in patients treated in intervention vs comparison group hospitals. Models controlled for variables in the Table and the percentage of non-Hispanic White individuals in the community and included hospital and time fixed effects. Error bars represent 95% CIs, calculated with SEs clustered on hospital and patient zip code. MOUD indicates medication for opioid use disorder.

Figure 3 shows estimates from event study analyses examining the association of OORP implementation with the 4 outcomes. The estimates corresponding to each half-year reflect changes in the measures relative to the half-year of program implementation. In the half-year after implementation, the OORP was associated with a 3.4–percentage point increase in the probability of 60-day MOUD initiation (0.034; 95% CI, 0.004-0.064), representing a 45% increase above the mean probability of 60-day MOUD initiation of 0.075 (95% CI, 0.066-0.084) during the preimplementation period, but there was no difference in later half-years. After OORP implementation, there was no change in the probability of psychosocial treatment initiation within 60 days at any of the time points. Implementation of the OORP was associated with a decrease in the number of medically treated overdoses within 180 days of −0.086 (95% CI, −0.154 to −0.018) in the fourth half-year and −0.106 (95% CI, −0.184 to −0.028) in the fifth half-year after implementation, representing 38% and 46% decreases, respectively. There was no association between OORP implementation and a change in all-cause acute care visits in the 180 days after the index overdose. Results from the sensitivity analyses were generally similar except there were no differences between MOUD initiation and repeat overdoses when using the half-year before OORP implementation as the reference period (eTables 3-11 in Supplement 1).

**Figure 4.**
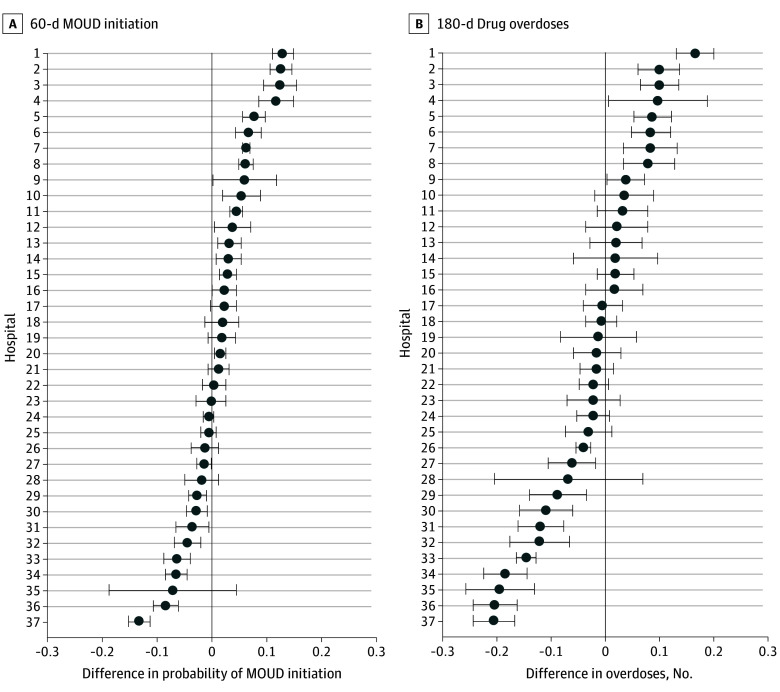
Associations of Opioid Overdose Recovery Program Implementation With 60-Day Initiation of Medication for Opioid Use Disorder (MOUD) and 180-Day Drug Overdoses by Hospital Treatment effects were estimated from 2 × 2 difference-in-differences models for 37 hospitals with 30 or more overdoses over the study period. Models controlled for variables in the Table. Differences in outcomes before and after OORP implementation in patients treated in the intervention hospitals vs the comparison group hospitals are shown. Error bars represent 95% CIs, calculated with SEs clustered on hospital and patient zip code.

Figure 4 shows results from 2 × 2 DID analyses conducted separately for the 37 hospitals with at least 30 observations over the study period. Differences in outcomes before and after OORP implementation among patients treated in the intervention hospitals vs the comparison group hospitals were analyzed. We found considerable heterogeneity across hospitals; program implementation was positively associated with 60-day MOUD initiation in 17 hospitals, with increases in probability ranging from 0.017 (95% CI, 0.006-0.027) to 0.130 (95% CI, 0.111-0.150) (Figure 4A). Four hospitals had differences greater than 0.1, meaning that the difference in probability of initiating an MOUD following program implementation was at least 10 percentage points higher for an average patient treated in the intervention hospitals compared with an average patient treated in the comparison group hospitals. Program implementation was negatively associated with 60-day MOUD in 8 hospitals, and for 12 hospitals, we found no association of implementation with MOUD initiation.

We found substantial variation across hospitals in the association between OORP implementation and the 180-day overdose outcome (Figure 4B). Implementation was associated with a greater decrease in overdoses among patients in the intervention group than among patients in the comparison group for 11 hospitals (30%), with decreases in the number of overdoses ranging from −0.205 (95% CI, −0.243 to −0.167) to −0.040 (95% CI, −0.054 to −0.026). There was no association between program implementation and a reduction in overdoses for 17 hospitals (46%), and for 9 hospitals (24%), overdoses increased compared with comparison group hospitals.

## Discussion

In this cohort study, OORP implementation was associated with higher probability of patients initiating an MOUD within 60 days and experiencing fewer medically treated overdoses within 180 days of discharge. However, we found no association between program implementation and changes in postdischarge psychosocial treatment initiation or all-cause acute care visits. Program outcomes varied based on time since implementation, with a significant increase in MOUD initiation found only in the first half-year after implementation and significant decreases in repeat overdose only during the fourth and fifth half-years after implementation. Estimated treatment effects were similar in sensitivity analyses using the half-year before implementation as the reference period but did not reach statistical significance. Analyses examining the program’s association with 60-day MOUD initiation and 180-day repeat overdoses separately for each hospital revealed considerable heterogeneity across sites, with some showing marked improvements and others having less favorable outcomes than comparison group sites. Collectively, these results suggest that implementation of a PRSS like the OORP may be associated with increased postdischarge treatment engagement and reduced repeat overdoses but that intervention outcomes likely vary based on implementation success and context.

These results add to prior research on outcomes of PRSSs for patients experiencing opioid overdose. Other studies have similarly shown that an ED-based PRSS may increase treatment engagement after discharge.^27,28,36,37,38^ Our finding that OORP implementation was associated with MOUD initiation but not with psychosocial treatment initiation may reflect limited availability of certain types of care,^29^ prioritization of MOUDs in the program design, or best practice guidelines recommending low-barrier MOUDs without requirements for psychosocial services.^39^ Findings for other PRSS outcomes have been less consistent. While we found an association of the OORP with reduced repeat overdose risk, other studies have had mixed results.^36,40,41^ Implementation of the OORP was not associated with reductions in all-cause acute care visits in this study, in contrast to another study showing a reduction in such visits among individuals receiving PRSSs.^42^ The inconsistent findings across studies may result from variations in PRSS program design and contexts (eg, program duration and staffing, additional services delivered concurrently).

Estimated program effects averaged across EDs may mask substantial effect estimates in specific sites. Accordingly, an important contribution of this study is that it demonstrated heterogeneity across EDs in the association of OORP implementation with MOUD initiation and repeat medically treated overdose. Whereas some intervention hospitals had large increases in patients initiating an MOUD or decreases in patients with repeat overdose compared with nonintervention sites, other intervention hospitals fared worse than their comparators. This heterogeneity may be driven by variations across sites in organizational structure, resources, relationships with hospitals and treatment agencies, and other factors described in qualitative research on this program.^29,31^ Programs may further vary in the challenges they face in implementing PRSSs and the diverging experiences of peers working in hospital settings. Implementation issues highlighted in prior research include high turnover,^43^ lack of program champions,^29,44^ and difficulties integrating peers into hospital hierarchical structures.^45,46^ As reported by peers in qualitative studies, challenges interfering with service delivery include role confusion,^47^ health care practitioner stigma,^45^ low awareness of peers’ roles,^48^ inadequate training and supervision,^47,48^ and burnout due to the work’s irregular schedule and emotionally taxing nature.^43,46^ Unmeasured patient-level factors (eg, readiness for change)^49^ and community-level factors (eg, treatment access and psychosocial resources) may further account for differences. Given the variation in implementation models for ED-based PRSSs, there is a need for further research aided by development of a fidelity measure to identify key intervention components of ED-based PRSSs and the factors influencing effectiveness.

Estimated program effects were also likely determined by unmeasured interventions to address OUD that were implemented in treatment or comparison group sites. For example, ED buprenorphine prescribing has been shown to be associated with increased postdischarge treatment engagement,^50,51,52^ and MOUDs have been associated with lower risk of overdose.^8,53,54^ In a study demonstrating that patients who received an ED-based intervention from a substance use navigator had greater treatment initiation, treatment initiation was increased further with ED-administered medication.^37^ Other evidence-based approaches, including addiction medicine consultations, social work interventions, and Screening, Brief Intervention, and Referral to Treatment also may have been implemented in study hospitals.^11,55^ Multicomponent strategies may have synergistic effects,^56^ and further research is needed to determine whether PRSSs on their own are sufficient for improving patient outcomes or whether they are more effective when implemented alongside other evidence-based strategies.

### Limitations

This study has several limitations. First, because we used deidentified data and the OORP was not a Medicaid-reimbursed service, we were unable to identify patients who received the OORP intervention. Our intention-to-treat design included patients who were not offered the program due to staff availability^29^ and patients who were approached but refused services,^57^ likely leading to conservative effect estimates. We were further unable to determine the level of service patients received, which ranged from a 1-time bedside intervention to 8 weeks of intensive support from a peer and case manager. This study only assessed outcomes observed in Medicaid data; treatment paid by other sources, overdoses not resulting in medical care, and mortality could not be identified. We could not determine whether other interventions to address OUD were implemented in study hospitals, which may have improved patient outcomes in comparison group hospitals or partially accounted for observed outcomes in intervention hospitals. Lastly, analyses used data from a single Medicaid program in a state with rapid expansion in OUD services^58,59^ and may not generalize to other states or payers with different policies, treatment access, and related contextual factors.

## Conclusions

In this cohort study of patients with opioid overdose, we found that OORP implementation was associated with an increase in MOUD initiation and a decrease in repeat overdoses. The large variation in findings across hospitals suggests that treatment outcomes are heterogeneous and may depend on higher-order implementation site factors, such as OORP implementation success, program embeddedness, and availability of other hospital-based OUD services.

## Notes

Supplement 1.
eMethods.
**eTable 1.** *ICD-9-CM* and *ICD-10-CM* Diagnosis Codes Used to Define Comorbidities **eTable 2.** National Drug Codes and Procedure Codes to Identify MOUD, Psychosocial SUD Treatment, and Health Services **eTable 3.** Event Study Model Estimates for the Association of OORP Implementation With Outcomes, With Half-Year Before Implementation as the Reference Period **eTable 4.** Association of OORP Implementation With Outcomes From 2 × 2 Difference-in-Differences Models **eTable 5.** Association of OORP Implementation With Outcomes From 2 × 2 Difference-in-Differences Models, by Implementation Wave **eTable 6.** Event Study Model Estimates for Association of OORP Implementation With Outcomes, Compared With Observations in Hospitals That Never Implemented OORP **eTable 7.** Two-Way Fixed-Effects Event Study Model Estimates for Association of OORP Implementation With Outcomes **eTable 8.** Event Study Model Estimates for Association of OORP Implementation With Outcomes Using Sun and Abraham Estimator **eTable 9.** Event Study Model Estimates for Association of OORP Implementation With Outcomes, Including Patients in Treatment in 30 Days Before Overdose **eTable 10.** Event Study Model Estimates for Association of OORP Implementation With Outcomes, Including All Patient Episodes During the Study Period **eTable 11.** Event Study Model Estimates for Association of OORP Implementation With Outcomes, Using Generalized Linear Models
eReferences

Supplement 2.
Data Sharing Statement
