# Supplementary material for: Emergency Department Peer Support Program and Patient Outcomes After Opioid Overdose
Source: JAMA Netw Open. 2024 Mar 25;7(3):e243614. doi: 10.1001/jamanetworkopen.2024.3614 (PMC10964115; doi:10.1001/jamanetworkopen.2024.3614)
Supplement: Supplement 1. — eMethods. eTable 1. ICD-9-CM and ICD-10-CM Diagnosis Codes Used to Define Comorbidities eTable 2. National Drug Codes and Procedure Codes to Identify MOUD, Psychosocial SUD Treatment, and Health Services eTable 3. Event Study Model Estimates for the Association of OORP Implementation With Outcomes, With Half-Year Before Implementation as the Reference Period eTable 4. Association of OORP Implementation With Outcomes From 2 × 2 Difference-in-Differences Models eTable 5. Association of OORP Implementation With Outcomes From 2 × 2 Difference-in-Differences Models, by Implementation Wave eTable 6. Event Study Model Estimates for Association of OORP Implementation With Outcomes, Compared With Observations in Hospitals That Never Implemented OORP eTable 7. Two-Way Fixed-Effects Event Study Model Estimates for Association of OORP Implementation With Outcomes eTable 8. Event Study Model Estimates for Association of OORP Implementation With Outcomes Using Sun and Abraham Estimator eTable 9. Event Study Model Estimates for Association of OORP Implementation With Outcomes, Including Patients in Treatment in 30 Days Before Overdose eTable 10. Event Study Model Estimates for Association of OORP Implementation With Outcomes, Including All Patient Episodes During the Study Period eTable 11. Event Study Model Estimates for Association of OORP Implementation With Outcomes, Using Generalized Linear Models eReferences [file jamanetwopen-e243614-s001.pdf]

Supplementary Online Content

Treitler P, Crystal S, Cantor J, et al. Emergency department peer support program and patient outcomes after opioid overdose. *JAMA Netw Open*. 2024;7(3):e243614. doi:10.1001/jamanetworkopen.2024.3614

eMethods

eTable 1. *ICD-9-CM* and *ICD-10-CM* Diagnosis Codes Used to Define Comorbidities

eTable 2. National Drug Codes and Procedure Codes to Identify MOUD, Psychosocial SUD Treatment, and Health Services

eTable 3. Event Study Model Estimates for the Association of OORP Implementation With Outcomes, With Half-Year Before Implementation as the Reference Period

eTable 4. Association of OORP Implementation With Outcomes From 2 × 2 Difference-in-Differences Models

eTable 5. Association of OORP Implementation With Outcomes From 2 × 2 Difference-in-Differences Models, by Implementation Wave

eTable 6. Event Study Model Estimates for Association of OORP Implementation With Outcomes, Compared With Observations in Hospitals That Never Implemented OORP

eTable 7. Two-Way Fixed-Effects Event Study Model Estimates for Association of OORP Implementation With Outcomes

eTable 8. Event Study Model Estimates for Association of OORP Implementation With Outcomes Using Sun and Abraham Estimator

eTable 9. Event Study Model Estimates for Association of OORP Implementation With Outcomes, Including Patients in Treatment in 30 Days Before Overdose

eTable 10. Event Study Model Estimates for Association of OORP Implementation With Outcomes, Including All Patient Episodes During the Study Period

eTable 11. Event Study Model Estimates for Association of OORP Implementation With Outcomes, Using Generalized Linear Models

eReferences

This supplementary material has been provided by the authors to give readers additional information about their work.

© 2024 Treitler P et al. *JAMA Network Open*.

Detailed Outcome and Measure Specifications

Outcomes

60-day MOUD initiation: Receipt of any MOUD within 60 days following discharge from an ED or inpatient visit. Outcome is a binary variable where 1 = yes and 0 = no. MOUD was identified using National Drug Codes (NDCs) on prescription claims and medication dispensing events documented as procedure codes on medical claims (eTable 2). For this outcome, patients were excluded from analyses using this outcome if they had any MOUD supply in the 30 days before the index overdose (see cohort flow diagram in manuscript Figure 1).

60-day psychosocial treatment initiation: Psychosocial treatment includes services corresponding to American Society of Addiction Medicine (ASAM) levels of care (i.e., outpatient, residential) excluding withdrawal management.[1] Treatment was identified using procedure codes shown in eTable 2 and required an SUD diagnosis on the claim. Outcome is a binary variable where 1 = yes and 0 = no. Patients were excluded from analyses using this outcome if they had any SUD treatment (psychosocial or MOUD) in the 30 days before the index overdose (see cohort flow diagram in manuscript Figure 1).

180-day overdoses: Overdoses are limited to those treated in ED or inpatient hospital settings; overdoses that did not result in a hospital visit (e.g., those in which naloxone was administered by emergency medical services but patients refuse further medical care, or those in which bystanders administered naloxone) are excluded. Drug overdoses were identified using diagnosis codes for any drug poisoning, as shown in eTable 1. Outcome is a continuous variable representing the number of overdoses occurring within 180 days.

180-day all-cause acute care visits: All-cause acute care (ED and inpatient) visits were identified using procedure and revenue codes (eTable 2) and were measured as the number of visits occurring within 180 days.

Adjustment Variables

Patient level:

Demographics: Demographics were obtained from Medicaid enrollment files. Patient age was categorized as 18-24, 25-34, 35-44, 45-54, 55-64. Sex was categorized as female and male. For race and ethnicity, we used a variable in the Medicaid data with categories of American Indian, Black, Latin, Oriental, White, Indo-Chinese/Refugee/Cubans Admitted After 9/30/78, SSA Classification, Cuban/Haitian (Refugee Act of 1980), Other, and Unknown. Responses were classified by individuals completing applications or by case workers assisting individuals with these applications. For the purposes of these analyses and to adjust for patient race and ethnicity (an important determinant of outcomes due to racism and other factors), we categorized the original categories into Black, Hispanic, White, and Other/unknown. Other/unknown includes the following categories: American Indian, Oriental, Indo-Chinese/Refugee/Cubans Admitted After 9/30/78, SSA Classification, Cuban/Haitian (Refugee Act of 1980), Other, and Unknown.

Comorbidities: We assessed comorbid diagnoses in the 180-day period before the index overdose. Patients were considered to have the diagnosis if they had 1 or more claims with a diagnostic code corresponding to the category. Comorbidities assessed were: opioid use disorder, other substance use disorder, any psychiatric disorder, hepatitis C, HIV/AIDS, and chronic pain. ICD-9-CM and ICD-10-CM codes for these diagnostic categories are in eTable 1. We also included an indicator of overall medical comorbidity by including a count of Chronic Conditions Warehouse[2] comorbidities (excluding conditions separately modeled) and categorized the variable into 0, 1-2, or 3+.

Prior health service use: We assessed health service use in the 180-day period before the index overdose. Health services include any inpatient visit, any ED visit, non-opioid drug overdose, any MOUD use, and any psychosocial service use. Psychosocial services were only included if they had

© 2024 Treitler P et al. *JAMA Network Open*.

an SUD diagnosis on the claim. Note that in analyses of treatment initiation, MOUD use and psychosocial service use are assessed in the 31-180 days before the index event, because individuals in treatment in the prior 30 days were excluded. Diagnosis and procedure codes for prior health service utilization are shown in eTable 1 and eTable 2.

Index visit characteristics: We controlled for characteristics of the index opioid overdose episode by including indicators of overdose from heroin or synthetic opioids vs. other opioids only, opioid overdoses that also involved non-opioid drugs, whether the ED visit led to an inpatient stay, and among those with inpatient stays, whether the stay included detoxification or psychiatric care. Diagnosis and procedure codes for index visit characteristics were identified on claims associated with the index ED visit and are shown in eTable 1 and eTable 2.

Community level:

Community characteristics were assessed at the zip code level, by linking patient zip code from enrollment files to publicly-available census and treatment availability data. The following measures were included:

Community sociodemographic/economic characteristics: The percentage of White, non-Hispanic residents and the Social Deprivation Index (SDI), a composite of 17 socioeconomic measures,[3] were calculated using the U.S. Census Bureau's 2015-2019 American Community Survey 5-year estimates.[4] The SDI contains measures related to income, employment, education, housing, and family characteristics.

Treatment access: We included annual measures of community treatment availability by calculating the number of specialty addiction treatment facilities and opioid treatment programs within 5 miles of patient zip codes, using address and provider type from SAMHSA's National Directory of Drug and Alcohol Abuse Treatment Facilities.[5] As a measure of buprenorphine access, we used Medicaid claims linked to the NPPES NPI file[6] containing provider addresses to calculate annual numbers of Medicaid-enrolled practitioners who prescribed buprenorphine at least once during the year within 5 miles of patient zip codes.

Hospital Attribution Methodology

Emergency department (ED) and inpatient claims separated by 1 day or less were combined into a single episode, since the majority of such instances are part of the same hospital visit that lasted more than one day. Even visits that last only one day typically generate many claims (e.g., pharmacy, ED or inpatient, professional claims from physicians or other service providers), so a hierarchical attribution methodology was developed to attribute patients to the correct facility for the visit.

First, each claim was given a score based on the provider type listed on the claim, identified using information from the NPPES NPI registry, linked by NPI number, as well as provider information in claims. Scores were as follows:

- 99: Claim is for an ED visit; provider type is acute care hospital
- 98: Claim is not for an ED visit; provider type is acute care hospital
- 97: Claim is for an ED visit; provider type is physician
- 96: Claim is for an ED visit; provider type is advanced practitioner
- 95: Claim is not for an ED visit; provider type is physician or advanced practitioner
- 94: Claim is not for an ED visit; provider type is hospital other than general acute care hospital
- 93: Claim is not for an ED visit; provider type is not physician or advanced practitioner
- 90: All other claims
-

For purposes of attribution, the claim with the highest score was selected and when there was more than 1 with the same score, the earlier claim was selected (e.g., if there were claims for ED visit with provider type acute care hospital on subsequent days).

© 2024 Treitler P et al. *JAMA Network Open*.

Using the address information associated with the claim (determined through linkage with the NPPES NPI file), the claim was attributed to one of 70 NJ acute care hospitals, by matching to addresses listed in a state Department of Health database.

Detailed Analytic Strategy

In descriptive analyses shown in manuscript Figure 2, we calculated unadjusted means in outcomes for intervention and comparison groups before and after OORP implementation. This was done by separately calculating these 4 means for each of the 7 OORP implementation waves, then aggregating them using the inverse variance method.[7]

For the main analysis, we implemented the “stacked regression” approach, described by Baker et al.[8] and Cengiz et al.[9] This method creates separate event-specific datasets using only “clean” controls (i.e., no “bad comparisons”),[10] attaches a dataset specific identifier variable to them, then stacks the datasets together. A two-way fixed effects event study regression is then estimated on the stacked dataset with dataset specific unit and time fixed effects. This procedure estimates event studies from each of the separate datasets, then combines treatment effects across cohorts using variance weighting.

When data are aggregated to half-year (6 months periods), there are 7 OORP implementation waves – that is, 7 groups of OORP hospitals that implemented during a specific half-year period. The 53 OORP hospitals implemented the program in 7 different half-year periods are shown in the table:

Half-year of implementation	# Hospitals implemented
1st half 2016	8
2nd half 2016	4
1st half 2017	13
2nd half 2017	17
1st half 2018	9
2nd half 2018	1
2nd half 2019	1

The analysis uses the following event study model:

$$y_{iht} = \gamma_h + \lambda_t + \sum_{\tau=-K_h}^{-1} \beta_{-\tau} DD_{h,t-\tau} + \sum_{\tau=1}^{L_h} \beta_\tau DD_{h,t+\tau} + X_{iht}\delta + \varepsilon_{iht}$$

In this model, Y_{iht} represents the outcome (e.g., treatment initiation) for individual *i* treated at hospital *h* in half-year *t*. γ_h and λ_t are fixed effects for hospital and half-year, respectively. The lead and lag terms are represented by the left and right summations, respectively. Separate terms are included for periods τ relative to the implementation half-year of $\tau = 0$, with K_h($-K_h$, …, -3, -2, -1) leads before OORP implementation and L_h (+1, +2, +3, … L_h) lags after OORP implementation. Separate B_τ’s are estimated for each half-year period τ, whose effects are interpreted relative to the excluded time period ($\tau = 0$ is used in this example). $DD_{h,t-\tau}$ *and* $DD_{h,t+\tau}$ are equal to 1 if hospital *h* implemented OORP and is t -/+ τ periods before or after implementation, and 0 otherwise. The estimates of interest are B_τ’s, which represent average effects of being treated for each τ. Negative values of τ represent pre-trends (commonly used to test the parallel trends assumption) and positive values of τ represent the treatment effect τ time periods after OORP implementation. This model allows for *dynamic* treatment effects where the effect of treatment changes over time, in contrast to a standard difference-in-differences model which estimates only one post-intervention treatment effect. In addition to these terms, the model also includes X_{iht}, a vector of individual (e.g., demographic and enrollment characteristics, comorbidities, and service use history) and community (e.g., social deprivation index, demographic characteristics, treatment availability) level controls. ε_{iht} represents an error term clustered at the hospital and community level.

Models were estimated using the *feglm* function in the R *fixest* package. Importantly, patients were only included in the comparison group if they were treated in hospitals that did not yet or never implemented OORP, thereby avoiding the "bad comparisons" problem described by Goodman-Bacon.[10]

The following hypothetical example illustrates this approach:

Illustration of treatment and comparison groups in the event study design

Hospital	Treatment Status as of 1st half 2018	1st half 2016	2nd half 2016	1st half 2017	2nd half 2017	1st half 2018	2nd half 2018	1st half 2019	2nd half 2019
		τ = -4	τ = -3	τ = -2	τ = -1	τ = 0	τ = 1	τ = 2	τ = 3
Hospital 1	Treated	No	No	No	No	Yes	Yes	Yes	Yes
Hospital 2	Treated	No	No	No	No	Yes	Yes	Yes	Yes
Hospital 3	Not-yet	No	No	No	No	No	Yes	Yes	Yes
Hospital 4	Not-yet	No	No	No	No	No	Yes	Yes	Yes
Hospital 5	Not-yet	No	No	No	No	No	No	Yes	Yes
Hospital 6	Not-yet	No	No	No	No	No	No	Yes	Yes
Hospital 7	Never	No	No	No	No	No	No	No	No
Hospital 8	Never	No	No	No	No	No	No	No	No

In this event study analysis, patients treated in Hospital 1 and Hospital 2 are in the treatment group, and patients treated in the remaining hospitals comprise the comparison group. Orange cells are half-year periods when hospitals did not have OORP and green cells are half-year periods when they did. Patients treated in hospitals 3-8 are in the comparison group at τ = 0 (the half-year of OORP implementation), whereas only patients treated in Hospital 7 and Hospital 8 are in the comparison group at τ = 3.

The following is an adaptation of the formula on page 2 to this hypothetical example:

$$y_{iht} = \gamma_h + \lambda_t + \beta_{-4}DD_{ht} + \beta_{-3}DD_{ht} + \beta_{-2}DD_{ht} + \beta_{-1}DD_{ht} + \beta_1 DD_{ht} + \beta_2 DD_{ht} + \beta_3 DD_{ht} + X_{iht}\delta + \varepsilon_{iht}$$

Note the t = 0 term is dropped to avoid perfect multicollinearity, since the $B_\tau DD_{st}$'s are dummy variables. Using the outcome of MOUD initiation within 60 days of overdose in a linear probability model, Y_{ist} represents the probability of initiating treatment. The B_τ's are the estimates of interest. If B_{-4}, B_{-3}, B_{-2}, and B_{-1} are not significantly different from 0, the parallel trends assumption is supported (but not definitively met), since they are relative to B_0, the excluded half-year of OORP implementation. B_1 is the OORP effect in the 1st half-year of implementation and B_3 is the effect in the 3rd half-year following implementation.

Sensitivity Analyses

First, we ran models that utilized the half-year before program implementation (HY = -1) as the reference period. Second, we assessed overall program effects in a 2x2 difference-in-differences analysis by aggregating observations into pre-and post-implementation periods, as well as separately for each implementation wave. Third, we tested models in which the comparison group was limited to overdoses in never-treated hospitals, excluding observations in not-yet-treated hospitals. Fourth, as alternatives to the stacked regression estimator, we fit two-way fixed effects (TWFE) models and used an estimator described by Sun and Abraham,[41] which avoids potential biases in TWFE models but only permits never-treated units in the comparison group. Fifth, for treatment initiation outcomes, we performed a sensitivity analysis in which patients in treatment in the 30 days before the index overdose were included. Sixth, we ran models that included all patient episodes during the study period, rather than the first episode only. Finally, we assessed the program association with outcomes using generalized linear models (binomial regression for binary outcomes and quasi-Poisson regression for count outcomes).

© 2024 Treitler P et al. *JAMA Network Open*.

eTable 1. ICD-9-CM and ICD-10-CM diagnosis codes used to define comorbidities

Category	Disorder	ICD-9-CM codes	ICD-10-CM codes
Drug Poisoning	Opioids	Heroin: 96501, E8500<br>Other opioid: 96500, 96502, 96509, 9701, E8501, E8502, E9350, E9351, E9352, E9401	Heroin or synthetic opioid: T401X1, T401X2, T401X3, T401X4, T404X1, T404X2, T404X3, T404X4, T404X5, T40411, T40412, T40413, T40414, T40415, T40421, T40422, T40423, T40424, T40425, T40491, T40492, T40493, T40494, T40495,<br>Other opioid:<br>T400X1, T400X2, T400X3, T400X4, T400X5, T402X1, T402X2, T402X3, T402X4, T402X5, T403X1, T403X2, T403X3, T403X4, T403X5, T40601, T40602, T40603, T40604, T40605, T40691, T40692, T40693, T40694, T40695
	Alcohol	9800, E8600, E8601	T510
	Cocaine	97081	T405X1, T405X2, T405X3, T405X4
	Cannabis and derivatives	N/A	T407
	Hallucinogens	E8541, 9696	T408, T409
	Benzodiazepines, sedatives, hypnotics	967, 9694, E851, E852, E8532	T42
	Psychostimulants	9697, E8542, E8543	T436
Substance use disorders	Opioid use disorder	30400, 30401, 30402, 30470, 30471, 30472, 30550, 30551, 30552	F1110, F11120, F11121, F11122, F11129, F1114, F11150, F11151, F11159, F11181, F11182, F11188, F1119, F1120, F11220, F11221, F11222, F11229, F1123, F1124, F11250, F11251, F11259, F11281, F11282, F11288, F1129, F1190, F11920, F11921, F11922, F11929, F1193, F1194, F11950, F11951, F11959, F11981, F11982, F11988, F1199
	Alcohol use disorders	2910, 2911, 2912, 2913, 2914, 2915, 29181, 29182, 29189, 2919, 30300, 30301, 30302, 30390, 30391, 30392, 30500, 30501, 30502, 3575, 4255, 53530, 53531, 5710, 5711, 5712, 5713	F101, F1010, F1012, F10120, F10121, F10129, F1014, F1015, F10150, F10151, F10159, F1018, F10180, F10181, F10182, F10188, F1019, F102, F1020, F1022, F10220, F10221, F10229, F1023, F10230, F10231, F10232, F10239, F1024, F10240, F1025, F10250, F10251, F10259, F1026, F1027, F1028, F10280, F10281, F10282, F10288, F1029
	Cannabis use disorder	30430, 30431, 30432, 30520, 30521, 30522	F121, F1210, F1212, F12120, F12121, F12122, F12129, F1215, F12150, F12151, F12159, F1218, F12180, F12188, F1219, F122, F1220, F1222, F12220, F12221, F12222, F12229, F1223, F12230, F1225, F12250, F12251, F12259, F1228, F12280, F12288, F1229

© 2024 Treitler P et al. *JAMA Network Open*.

Category	Disorder	ICD-9-CM codes	ICD-10-CM codes
	Sedative, hypnotic, anxiolytic use disorders	30410, 30411, 30412, 30540, 30541, 30542	F131, F1310, F1312, F13120, F13121, F13129, F1314, F1315, F13150, F13151, F13159, F1318, F13180, F13181, F13182, F13188, F1319, F132, F1320, F1322, F13220, F13221, F13229, F1323, F13230, F13231, F13232, F13239, F1324, F13240, F1325, F13250, F13251, F13259, F1326, F1327, F1328, F13280, F13281, F13282, F13288, F1329
	Stimulant and cocaine use disorders	30420, 30421, 30422, 30560, 30561, 30562, 30440, 30441, 30442, 30570, 30571, 30572	F141, F1410, F1412, F14120, F14121, F14122, F14129, F1414, F1415, F14150, F14151, F14159, F1418, F14180, F14181, F14182, F14188, F1419, F142, F1420, F1422, F14220, F14221, F14222, F14229, F1423, F14230, F1424, F14240, F1425, F14250, F14251, F14259, F1428, F14280, F14281, F14282, F14288, F1429, F151, F1510, F1512, F15120, F15121, F15122, F15129, F1514, F1515, F15150, F15151, F15159, F1518, F15180, F15181, F15182, F15188, F1519, F152, F1520, F1522, F15220, F15221, F15222, F15229, F1523, F15230, F1524, F15240, F1525, F15250, F15251, F15259, F1528, F15280, F15281, F15282, F15288, F1529
	Other substance use disorders (hallucinogens, inhalants, other, and unspecified)	30450, 30451, 30452, 30530, 30531, 30532, 30460, 30461, 30462, 30480, 30481, 30482, 30490, 30491, 30492, 30590, 30591, 30592	F161, F1610, F1612, F16120, F16121, F16122, F16129, F1614, F1615, F16150, F16151, F16159, F1618, F16180, F16183, F16188, F1619, F162, F1620, F1622, F16220, F16221, F16229, F1624, F16240, F1625, F16250, F16251, F16259, F1628, F16280, F16283, F16288, F1629, F181, F1810, F1812, F18120, F18121, F18129, F1814, F1815, 18150, F18151, F18159, F1817, F18170, F1818, F18180, F18188, F1819, F182, F1820, F1822, F18220, F18221, F18229, F1824, F18240, F1825, F18250, F18251, F18259, F1827, F1828, F18280, F18288, F1829, F191, F1910, F1912, F19120, F19121, F19122, F19129, F1914, F1915, F19150, F19151, F19159, F1916, F19160, F1917, F19170, F1918, F19180, F19181, F19182, F19188, F1919, F192, F1920, F1922, F19220, F19221, F19222, F19229, F1923, F19230, F19231, F19232, F19239, F1924, F19240, F1925, F19250, F19251, F19259, F1926, F1927,

© 2024 Treitler P et al. *JAMA Network Open*.

Category	Disorder	ICD-9-CM codes	ICD-10-CM codes
			F1928, F19280, F19281, F19282, F19288, F1929
Psychiatric disorders	Schizophrenia	295, 297, 2983, 2984, 2988, 2989, 30122	F20, F21, F22, F23,F24, F25, F28, F29
	Bipolar disorder and manic affective disorder	2960, 2961, 2964, 2965, 2966, 2967, 2968	F30, F31, F340, F3481
	Depression and other mood disorders not included under bipolar	29620, 29621, 29622, 29623, 29624, 29625, 29626, 29630, 29631, 29632, 29633, 29634, 29635, 29636, 2980, 311, 29682, 29690, 29699, 30110, 30112, 30113, 3004	F32, F33, F341, F3489, F39;
	Anxiety and OCD	3000, 3002, 3003	F40, F41, F42
	PTSD and acute stress	308, 30981	F430, F431
	Adjustment disorders and severe stress reaction	3090, 3091,3092, 3093, 3094, 30982,30983, 30989, 3099	F432, F438, F439
	Personality, impulse control, sexual disorders	301, 302, 3123, 30016, 30019, 3019	F60, F63, F64, F65, F66, F68, F69
	Other psychiatric disorders	29420, 29421, 2982, 3001, 3008, 306, 3007, 30780, 30789, 3006, 31081, 3005, 30089, 3009, V402, V409, 3071, 30750, 30751, 30754, 30759, 30740, 30741, 30742, 30744, 32702, 30746, 30747, 30749, 32715, 32719, 30270, 30271, 30272, 30273, 30274, 30275, 30276, 30279, 30651, V417, 29389, 3068, 31531, 31534, 31535, 31539, 31500, 31502, 31509, 3151, 3152, 3159, V400, 3154, 29900, 29901, 29910, 29980, 29981, 29990, 3308, 3158, 3159, 314, 3120, 3121, 3122, 3128, 3129, 31381, 30921, 3130, 3131, 31321, 31322, 3133, 31382, 31383, 31389, 3139, 31323, 31389, 30929, 3139, 3072, 3076, 3077, 30752,	F0390, F0391, F44, F45, F48, F50, F51, F52, F53, F59, F80, F81, F82, F84, F88, F89, F90, F91, F93, F94, F95, F98, F99

© 2024 Treitler P et al. *JAMA Network Open*.

Category	Disorder	ICD-9-CM codes	ICD-10-CM codes
		30753, 30759, 3070, 3073, 31389, 3139, 3009	
Medical comorbidities	Hepatitis C	07041, 07044, 07051, 07054, 07070, 07071, V0262	B1710, B1711, B182, B1920, B1921, K732, Z2252
	HIV/AIDS	042, 07953, V08	B20, B9735, O98711, O98712, O98713, O98719, O9872, O9873, Z21
	Chronic pain	30781, 3370, 3371, 3380, 3382, 3384, 339, 346, 3502, 3540, 3544, 355, 356, 357, 377, 710, 711, 712, 713, 714, 715, 716, 717, 718, 719, 720, 721, 722, 723, 724, 725, 726, 727, 728, 729, 730, 731, 732, 733, 734, 735, 736, 737, 738, 739, 7840	E0842, E0942, E1042, E1142, E1342, G43, G44, G501, G560, G564, G57, G589, G60, G61, G62, G63, G64, G65, G890, G892, G894, G900, G990, H46, H47, M00, M01, M02, M05, M06, M07, M08, M11, M12, M13, M14, M15, M16, M17, M18, M19, M20, M21, M22, M23, M24, M25, M30, M31, M32, M33, M34, M35, M36, M37, M38, M39, M40, M41, M42, M43, M44, M45, M46, M47, M48, M49, M50, M51, M52, M53, M54, M55, M56, M57, M58, M59, M60, M61, M62, M63, M64, M65, M66, M67, M68, M69, M70, M71, M72, M73, M74, M75, M76,M77, M78, M79, M80, M81, M82, M83, M84, M85, M86, M87, M88, M89, M90, M91, M92, M93, M94, M95, M96, M97, M98, M99, R262, R294, R29898, R51

© 2024 Treitler P et al. *JAMA Network Open*.

eTable 2. National Drug Codes and procedure codes to identify MOUD, psychosocial SUD treatment, and health services

Medication or service	National drug codes	Procedure or revenue codes
Buprenorphine	00378876516, 00378876593, 00378876616, 00378876693, 00378876716, 00378876793, 00378876816, 00378876893, 00781721606, 00781721664, 00781722706, 00781722764, 00781723806, 00781723864, 00781724906, 00781724964, 12496120201, 12496120203, 12496120401, 12496120403, 12496120801, 12496120803, 12496121201, 12496121203, 43598057901, 43598057930, 43598058001, 43598058030, 43598058101, 43598058130, 43598058201, 43598058230, 47781035503, 47781035511, 47781035603, 47781035611, 47781035703, 47781035711, 47781035803, 47781035811, 52427069203, 52427069211, 52427069403, 52427069411, 52427069803, 52427069811, 52427071203, 52427071211, 54569639900, 55700014730, 59385001201, 59385001230, 59385001401, 59385001430, 59385001601, 59385001630, 52440010014, 58284010014, 12496010001, 12496010002, 12496010005, 12496030001, 12496030002, 12496030005, 00054017613, 00054017713, 00054018813, 00054018913, 00093537856, 00093537956, 00093572056, 00093572156, 00228315303, 00228315403, 00228315473, 00228315503, 00228315567, 00228315573, 00228315603, 00378092393, 00378092493, 00406192303, 00406192403, 00406800503, 00406802003, 12496127802, 12496128302, 12496130602, 12496131002, 16590066630, 35356000407, 35356000430, 35356055530, 35356055630, 42291017430, 42291017530, 42858050103, 42858050203, 43063018407, 43063018430, 43063066706, 43063075306, 49999039507, 49999039515, 49999039530, 49999063830, 49999063930, 50268014411, 50268014415, 50268014511, 50268014515, 50383028793, 50383029493, 50383092493, 50383093093, 52959030430, 52959074930, 54123011430, 54123090730, 54123091430, 54123092930, 54123095730, 54123098630, 54569549600, 54569573900, 54569573901, 54569573902, 54569640800, 54868570700, 54868570701, 54868570702, 54868570703, 54868570704, 54868575000, 55045378403, 55700018430, 55700030230, 55700030330, 60429058611, 60429058630, 60429058633, 60429058711, 60429058730, 60429058733, 60687048111, 60687048121, 60687049211, 60687049221, 62175045232, 62175045832, 62756045983, 62756046083, 62756096983,	H0020HF26*, H0033HF*, H0033HF26*, H0033HH*, Q9991, Q9992, J0570-J0575, H0018HFU2, H0019HFU2

© 2024 Treitler P et al. *JAMA Network Open*.

	62756097083, 63629402801, 63629403401, 63629403402, 63629403403, 63874108403, 63874108503, 63874117303, 65162041503, 65162041603, 66336001630, 68071138003, 68071151003, 68258299903, 68308020230, 68308020830, 71335115403	
Methadone	N/A – only billed using procedure codes specific to opioid treatment programs	H0020, H0020HF,* H0020HF26,* Z2006,* Z3357,* H0018HFU1,* H0019HFU1*, S0109
Naltrexone	00056001122, 00056001130, 00056001170, 00056007950, 00056008050, 00185003901, 00185003930, 00406009201, 00406009203, 00406117001, 00406117003, 00555090201, 00555090202, 00904703604, 16729008101, 16729008110, 42291063230, 43063059115, 47335032683, 47335032688, 50090286600, 50436010501, 51224020630, 51224020650, 51285027501, 51285027502, 52152010502, 52152010504, 52152010530, 54868557400, 63459030042, 63629104601, 63629104701, 65694010003, 65694010010, 65757030001, 65757030202, 68084029111, 68084029121, 68094085362, 68115068030	J2315
Psychosocial SUD treatment (required SUD diagnosis on claim)	N/A	90832, 90833, 90834, 90836, 90837, 90838, 90839, 90840, 90845, 90846, 90847, 90849, 90853, 90875, 90876, H0004, H0005, H0015, H0018, H0019, H0035, H2001, H2012, H2013, H2034, H2035, H2036, G0155, G0176, G0410, G0411, Z2000, Z2001, Z2002, Z2003, Z2004, Z2005, Z2007, Z3335, Z3336, Z3337, Z3338, Z3339, Z3343, Z3344, Z3345, Z3346, Z3347, Z3348, Z3349, Z3354, Z3355, Z3356, Z3358, T1006, T1015HE, T1041HEHH, T1041HFHH, S0201, S9480, G0473, OP912, OP913, OP914, OP915
Inpatient stay	N/A	Inpatient revenue codes: 100, 101, 110, 111, 112, 113, 114, 116, 117, 118, 119, 120, 121, 122, 123, 124,126, 127, 128, 129, 130, 131, 132, 133, 134, 136, 137, 138, 139, 140, 141, 142, 143, 144, 146, 147, 148, 149, 150, 151,

© 2024 Treitler P et al. *JAMA Network Open*.

		152, 153, 154, 156, 157, 158, 159, 160, 164, 167, 169, 170, 171, 172, 173, 174, 179, 190, 191, 192, 193, 194, 199, 200, 201, 202, 203, 204, 206, 207, 208, 209, 210, 211, 212, 213, 214, 219, 1000, 1001, 1002
Inpatient detox	N/A	Inpatient revenue codes: 114, 124, 134, 144, 154
Inpatient psychiatric care	N/A	Inpatient revenue codes: 116, 126, 136, 146, 156
Emergency department visit	N/A	Inpatient or outpatient revenue codes: 450, 451, 452, 453, 454, 455, 456, 457, 458, 459, 981<br>CPT procedure codes: 99281, 99282, 99283, 99284, 99285

*H0020HF26 was used for buprenorphine dispensed in an opioid treatment program before July 2016. Starting July 2016, H0033HF, H0033HF26, and H0033HH were used.

© 2024 Treitler P et al. *JAMA Network Open*.

eTable 3: Event study model estimates for the association of OORP implementation with outcomes, with half-year before implementation as the reference period

Half-year	60-day MOUD initiation	60-day psychosocial treatment initiation	180-day overdose	180-day all-cause acute care utilization
≤-6	-0.029 (-0.067, 0.009)	-0.014 (-0.040, 0.011)	0.016 (-0.030, 0.063)	0.280 (0.005, 0.554)
-5	-0.017 (-0.046, 0.011)	0.012 (-0.027, 0.052)	0.006 (-0.061, 0.073)	-0.021 (-0.372, 0.331)
-4	-0.025 (-0.060, 0.011)	0.010 (-0.026, 0.046)	0.013 (-0.061, 0.087)	0.291 (-0.115, 0.697)
-3	-0.018 (-0.051, 0.015)	0.009 (-0.029, 0.047)	-0.017 (-0.096, 0.061)	-0.105 (-0.456, 0.246)
-2	0.001 (-0.031, 0.031)	-0.012 (-0.046, 0.021)	0.024 (-0.039, 0.088)	0.297 (0.023, 0.570)
-1	REF	REF	REF	REF
0	-0.009 (-0.045, 0.028)	0.017 (-0.017, 0.052)	0.028 (-0.044, 0.101)	-0.040 (-0.295, 0.216)
1	0.025 (-0.004, 0.054)	0.004 (-0.031, 0.038)	-0.022 (-0.084, 0.040)	0.106 (-0.186, 0.397)
2	-0.001 (-0.035, 0.034)	0.000 (-0.035, 0.035)	0.039 (-0.041, 0.120)	0.176 (-0.105, 0.457)
3	0.003 (-0.039, 0.045)	-0.018 (-0.059, 0.024)	-0.023 (-0.097, 0.051)	0.107 (-0.261, 0.475)
4	-0.014 (-0.056, 0.029)	-0.023 (-0.070, 0.023)	-0.058 (-0.135, 0.020)	0.009 (-0.340, 0.357)
5	0.025 (-0.019, 0.068)	-0.019 (-0.080, 0.043)	-0.078 (-0.168, 0.012)	-0.078 (-0.473, 0.317)
≥6	0.035 (-0.029, 0.100)	0.008 (-0.046, 0.062)	-0.040 (-0.146, 0.066)	0.145 (-0.264, 0.554)

Half-years are relative to OORP implementation, which is represented as half-year 0. MOUD and psychosocial treatment effects are probabilities; overdose and acute care utilization effects are counts. Estimates represent the difference in outcomes relative to the half-year before OORP implementation (time = -1) in patients treated in intervention vs. comparison group hospitals. Models control for variables in Table 1, include hospital and time fixed effects. 95% confidence intervals in parentheses were calculated with standard errors clustered on hospital and patient zip code.

© 2024 Treitler P et al. *JAMA Network Open*.

eTable 4. Association of OORP implementation with outcomes from 2x2 difference-in-difference models

Outcome	Coefficient (95% CI)
60-day MOUD initiation	0.028 (0.006, 0.049)
60-day psychosocial treatment initiation	0.006 (-0.019, 0.031)
180-day drug overdoses	-0.037 (-0.082, 0.008)
180-day all-cause ED or inpatient visits	0.007 (-0.202, 0.217)

Models use stacked regression with all half-years before implementation (half-years -9 to -1) and all half-years after implementation (half-years 0 to 8) aggregated. Estimates represent the difference in outcomes after OORP implementation in patients treated in intervention vs. comparison group hospitals. MOUD and psychosocial treatment effects are probabilities; overdose and acute care utilization effects are counts. Models control for variables in Table 1, include hospital and time fixed effects. 95% confidence intervals in parentheses were calculated with standard errors clustered on hospital and patient zip code.

© 2024 Treitler P et al. *JAMA Network Open*.

eTable 5. Association of OORP implementation with outcomes from 2x2 difference-in-difference models, by implementation wave

Wave	60-day MOUD initiation	60-day psychosocial treatment initiation	180-day overdose	180-day all-cause acute care utilization
1	0.035<br>(0.002, 0.068)	0.023<br>(-0.194, 0.065)	-0.048<br>(-0.106, 0.011)	-0.062<br>(-0.479, 0.354)
2	-0.015<br>(-0.060, 0.030)	-0.017<br>(-0.070, 0.036)	0.055<br>(-0.001, 0.112)	-0.086<br>(-0.364, 0.192)
3	0.021<br>(-0.001, 0.043)	0.007<br>(-0.038, 0.052)	-0.056<br>(-0.129, 0.017)	0.098<br>(-0.330, 0.526)
4	0.018<br>(-0.010, 0.047)	-0.015<br>(-0.059, 0.029)	-0.029<br>(-0.092, 0.034)	0.128<br>(-0.140, 0.396)
5	0.010<br>(-0.042, 0.062)	-0.064<br>(-0.111, -0.017)	-0.045<br>(-0.114, 0.024)	0.002<br>(-0.308, 0.312)
6	0.139<br>(0.090, 0.188)	0.101<br>(0.041, 0.161)	0.008<br>(-0.077, 0.093)	-0.442<br>(-0.715, -0.170)
7	0.061<br>(-0.005, 0.127)	-0.084<br>(-0.139, -0.030)	0.136<br>(0.077, 0.196)	-0.351<br>(-0.591, -0.112)

Models use stacked regression with all half-years before implementation (half-years -9 to -1) and all half-years after implementation (half-years 0 to 8) aggregated for each wave. Estimates represent the difference in outcomes after OORP implementation in patients treated in intervention vs. comparison group hospitals for each wave. MOUD and psychosocial treatment effects are probabilities; overdose and acute care utilization effects are counts. Models control for variables in Table 1 and include hospital and time fixed effects. 95% confidence intervals in parentheses were calculated with standard errors clustered on hospital and patient zip code.

© 2024 Treitler P et al. *JAMA Network Open*.

eTable 6: Event study model estimates for association of OORP implementation with outcomes, compared with observations in hospitals that never implemented OORP

Half-year	60-day MOUD initiation	60-day psychosocial treatment initiation	180-day overdose	180-day all-cause acute care utilization
≤-6	-0.034<br>(-0.092, 0.023)	-0.047<br>(-0.113, 0.019)	-0.022<br>(-0.118, 0.074)	0.634<br>(0.101, 1.167)
-5	-0.015<br>(-0.058, 0.028)	-0.003<br>(-0.060, 0.054)	-0.033<br>(-0.125, 0.058)	0.263<br>(-0.202, 0.728)
-4	-0.023<br>(-0.064, 0.017)	-0.015<br>(-0.057, 0.027)	-0.023<br>(-0.100, 0.054)	0.384<br>(-0.076, 0.844)
-3	-0.018<br>(-0.053, 0.017)	-0.012<br>(-0.056, 0.032)	-0.049<br>(-0.117, 0.020)	-0.025<br>(-0.374, 0.324)
-2	-0.001<br>(-0.032, 0.034)	-0.031<br>(-0.055, 0.004)	-0.008<br>(-0.076, 0.059)	0.388<br>(0.118, 0.658)
-1	-0.002<br>(-0.038, 0.033)	-0.013<br>(-0.047, 0.022)	-0.033<br>(-0.096, 0.029)	0.015<br>(-0.212, 0.243)
0	REF	REF	REF	REF
1	0.306<br>(0.002, 0.059)	-0.006<br>(-0.039, 0.027)	-0.039<br>(-0.095, 0.017)	0.186<br>(-0.053, 0.426)
2	0.008<br>(-0.025, 0.040)	-0.015<br>(-0.050, 0.020)	0.018<br>(-0.038, 0.074)	0.210<br>(-0.055, 0.474)
3	0.003<br>(-0.038, 0.044)	-0.023<br>(-0.060, 0.015)	-0.043<br>(-0.108, 0.021)	0.163<br>(-0.134, 0.461)
4	-0.004<br>(-0.038, 0.030)	-0.033<br>(-0.081, 0.015)	-0.086<br>(-0.148, -0.023)	0.063<br>(-0.219, 0.344)
5	0.027<br>(-0.013, 0.067)	-0.028<br>(-0.087, 0.032)	-0.106<br>(-0.181, -0.031)	-0.050<br>(-0.392, 0.291)
≥6	0.040<br>(-0.026, 0.106)	-0.003<br>(-0.059, 0.053)	-0.081<br>(-0.194, 0.033)	0.153<br>(-0.250, 0.555)

Half-years are relative to OORP implementation, which is represented as half-year 0. MOUD and psychosocial treatment effects are probabilities; overdose and acute care utilization effects are counts. Estimates represent the difference in outcomes relative to the half-year of OORP implementation (time = 0) in patients treated in intervention vs. comparison group hospitals. Models control for variables in Table 1 and include hospital and time fixed effects. 95% confidence intervals in parentheses were calculated with standard errors clustered on hospital and patient zip code.

© 2024 Treitler P et al. *JAMA Network Open*.

eTable 7. Two-way fixed effects event study model estimates for association of OORP implementation with outcomes

Half-year	60-day MOUD initiation	60-day psychosocial treatment initiation	180-day overdose	180-day all-cause acute care utilization
≤-6	-0.020<br>(-0.073, 0.034)	-0.029<br>(-0.104, 0.047)	-0.017<br>(-0.094, 0.061)	0.417<br>(-0.096, 0.929)
-5	-0.006<br>(-0.044, 0.031)	-0.002<br>(-0.061, 0.057)	-0.018<br>(-0.095, 0.060)	0.127<br>(-0.319, 0.574)
-4	-0.014<br>(-0.054, 0.026)	-0.003<br>(-0.051, 0.045)	-0.012<br>(-0.088, 0.064)	0.361<br>(-0.057, 0.778)
-3	-0.007<br>(-0.046, 0.031)	-0.004<br>(-0.051, 0.044)	-0.041<br>(-0.102, 0.021)	-0.037<br>(-0.351, 0.277)
-2	0.009<br>(-0.023, 0.041)	-0.022<br>(-0.059, 0.015)	-0.008<br>(-0.077, 0.060)	0.315<br>(0.068, 0.562)
-1	0.007<br>(-0.027, 0.041)	-0.012<br>(-0.045, 0.021)	-0.021<br>(-0.086, 0.043)	0.076<br>(-0.132, 0.285)
0	REF	REF	REF	REF
1	0.033<br>(0.004, 0.061)	-0.003<br>(-0.034, 0.028)	-0.039<br>(-0.106, 0.028)	0.162<br>(-0.079, 0.402)
2	0.006<br>(-0.026, 0.037)	-0.001<br>(-0.036, 0.034)	0.009<br>(-0.048, 0.067)	0.218<br>(-0.041, 0.476)
3	0.007<br>(-0.031, 0.044)	-0.013<br>(-0.049, 0.023)	-0.037<br>(-0.095, 0.022)	0.168<br>(-0.132, 0.467)
4	-0.006<br>(-0.008, 0.069)	-0.022<br>(-0.066, 0.022)	-0.082<br>(-0.150, -0.015)	0.024<br>(-0.244, 0.292)
5	0.031<br>(-0.008, 0.069)	-0.012<br>(-0.066, 0.042)	-0.090<br>(-0.162, -0.018)	0.001<br>(-0.307, 0.308)
≥6	0.041<br>(-0.029, 0.111)	-0.004<br>(-0.061, 0.053)	-0.041<br>(-0.149, 0.067)	0.26<br>(-0.206, 0.617)

Half-years are relative to OORP implementation, which is represented as half-year 0. MOUD and psychosocial treatment effects are probabilities; overdose and acute care utilization effects are counts. Estimates represent the difference in outcomes relative to the half-year of OORP implementation (time = 0) in patients treated in intervention vs. comparison group hospitals. Models control for variables in Table 1 and include hospital and time fixed effects. 95% confidence intervals in parentheses were calculated with standard errors clustered on hospital and patient zip code.

© 2024 Treitler P et al. *JAMA Network Open*.

eTable 8. Event study model estimates for association of OORP implementation with outcomes using Sun and Abraham estimator

Half-year	60-day MOUD initiation	60-day psychosocial treatment initiation	180-day overdose	180-day all-cause acute care utilization
≤-6	-0.078 (-0.120, -0.036)	-0.096 (-0.161, -0.030)	0.021 (-0.063, 0.105)	0.360 (-0.093, 0.812)
-5	-0.028 (-0.072, 0.016)	-0.035 (-0.094, 0.024)	-0.021 (-0.110, 0.068)	0.135 (-0.359, 0.628)
-4	-0.024 (-0.060, 0.011)	-0.023 (-0.070, 0.024)	-0.009 (-0.097, 0.079)	0.437 (-0.047, 0.920)
-3	-0.018 (-0.049, 0.013)	-0.020 (-0.069, 0.029)	-0.033 (-0.121, 0.055)	-0.009 (-0.425, 0.406)
-2	0.000 (-0.029, 0.029)	-0.035 (-0.071, 0.000)	-0.009 (-0.079, 0.061)	0.376 (0.031, 0.721)
-1	0.007 (-0.023, 0.037)	-0.010 (-0.041, 0.021)	-0.039 (-0.092, 0.013)	-0.007 (-0.274, 0.259)
0	REF	REF	REF	REF
1	0.032 (0.001, 0.062)	-0.007 (-0.039, 0.025)	-0.048 (-0.104, 0.007)	0.168 (-0.086, 0.423)
2	0.015 (-0.015, 0.045)	-0.011 (-0.048, 0.026)	0.008 (-0.052, 0.068)	0.223 (-0.035, 0.481)
3	0.014 (-0.024, 0.051)	-0.020 (-0.061, 0.021)	-0.058 (-0.123, 0.006)	0.171 (-0.171, 0.514)
4	0.002 (-0.031, 0.035)	-0.033 (-0.080, 0.013)	-0.095 (-0.174, -0.016)	0.107 (-0.244, 0.458)
5	0.038 (-0.009, 0.084)	-0.029 (-0.089, 0.031)	-0.119 (-0.198, -0.039)	0.035 (-0.355, 0.424)
≥6	0.061 (-0.004, 0.126)	0.004 (-0.058, 0.066)	-0.093 (-0.206, 0.019)	0.120 (-0.340, 0.581)

Half-years are relative to OORP implementation, which is represented as half-year 0. MOUD and psychosocial treatment effects are probabilities; overdose and acute care utilization effects are counts. Estimates represent the difference in outcomes relative to the half-year of OORP implementation (time = 0) in patients treated in intervention vs. comparison group hospitals. Models control for variables in Table 1 and include hospital and time fixed effects. 95% confidence intervals in parentheses were calculated with standard errors clustered on hospital and patient zip code.

© 2024 Treitler P et al. *JAMA Network Open*.

eTable 9. Event study model estimates for association of OORP implementation with outcomes, including patients in treatment in 30 days before overdose

Time relative to implementation	60-day MOUD initiation	60-day psychosocial treatment initiation
≤-6	-0.008 (-0.044, 0.029)	-0.035 (-0.078, 0.008)
-5	-0.019 (-0.052, 0.014)	-0.015 (-0.063, 0.033)
-4	-0.022 (-0.061, 0.017)	-0.017 (-0.059, 0.024)
-3	-0.009 (-0.045, 0.028)	-0.006 (-0.050, 0.037)
-2	0.016 (-0.022, 0.054)	-0.028 (-0.072, 0.015)
-1	0.007 (-0.033, 0.046)	-0.018 (-0.056, 0.020)
0	REF	REF
1	0.035 (0.003, 0.067)	0.001 (-0.030, 0.033)
2	-0.005 (-0.043, 0.033)	-0.016 (-0.053, 0.022)
3	0.020 (-0.021, 0.060)	-0.023 (-0.066, 0.020)
4	0.009 (-0.024, 0.043)	-0.028 (-0.080, 0.024)
5	0.023 (-0.014, 0.061)	-0.013 (-0.070, 0.044)
≥6	0.065 (0.003, 0.126)	0.003 (-0.058, 0.063)

Half-years are relative to OORP implementation, which is represented as half-year 0. MOUD and psychosocial treatment effects are probabilities; overdose and acute care utilization effects are counts. Estimates represent the difference in outcomes relative to the half-year of OORP implementation (time = 0) in patients treated in intervention vs. comparison group hospitals. Models control for variables in Table 1 and include hospital and time fixed effects. 95% confidence intervals in parentheses were calculated with standard errors clustered on hospital and patient zip code.

© 2024 Treitler P et al. *JAMA Network Open*.

eTable 10. Event study model estimates for association of OORP implementation with outcomes, including all patient episodes during the study period

Half-year	60-day MOUD initiation	60-day psychosocial treatment initiation	180-day overdose	180-day all-cause acute care utilization
≤-6	-0.010<br>(-0.031, 0.011)	-0.019<br>(-0.063, 0.026)	-0.001<br>(-0.117, 0.115)	0.535<br>(0.078, 0.992)
-5	-0.005<br>(-0.034, 0.024)	0.009<br>(-0.032, 0.050)	0.074<br>(-0.123, 0.270)	0.265<br>(-0.493, 1.023)
-4	-0.020<br>(-0.057, 0.018)	-0.008<br>(-0.044, 0.027)	-0.028<br>(-0.142, 0.087)	0.333<br>(-0.249, 0.914)
-3	-0.010<br>(-0.043, 0.022)	0.014<br>(-0.020, 0.049)	-0.119<br>(-0.228, -0.010)	-0.401<br>(-0.797, -0.004)
-2	0.015<br>(-0.016, 0.045)	-0.003<br>(-0.038, 0.033)	-0.134<br>(-0.254, -0.014)	-0.159<br>(-0.534, 0.216)
-1	0.014<br>(-0.017, 0.045)	-0.004<br>(-0.035, 0.027)	-0.099<br>(-0.228, 0.031)	-0.173<br>(-0.543, 0.197)
0	REF	REF	REF	REF
1	0.031<br>(0.004. 0.059)	0.014<br>(-0.013, 0.041)	-0.069<br>(-0.153, 0.015)	-0.056<br>(-0.302, 0.191)
2	0.003<br>(-0.026, 0.032)	0.011<br>(-0.022, 0.045)	-0.074<br>(-0.161, 0.014)	-0.075<br>(-0.526, 0.375)
3	0.012<br>(-0.016, 0.041)	-0.002<br>(-0.035, 0.032)	-0.119<br>(-0.235, -0.002)	-0.106<br>(-0.513, 0.301)
4	-0.006<br>(-0.034, 0.021)	-0.019<br>(-0.064, 0.026)	-0.135<br>(-0.229, -0.040)	-0.088<br>(-0.491, 0.316)
5	0.015<br>(-0.018, 0.049)	-0.005<br>(-0.053, 0.044)	-0.177<br>(-0.287, -0.066)	-0.096<br>(-0.540, 0.347)
≥6	0.042<br>(-0.004, 0.088)	0.011<br>(-0.040, 0.062)	-0.198<br>(-0.402, 0.006)	-0.232<br>(-0.792, 0.328)

Half-years are relative to OORP implementation, which is represented as half-year 0. MOUD and psychosocial treatment effects are probabilities; overdose and acute care utilization effects are counts. Estimates represent the difference in outcomes relative to the half-year of OORP implementation (time = 0) in patients treated in intervention vs. comparison group hospitals. Models control for variables in Table 1 and include hospital and time fixed effects. 95% confidence intervals in parentheses were calculated with standard errors clustered on hospital, patient zip code, and patient ID.

© 2024 Treitler P et al. *JAMA Network Open*.

eTable 11. Event study model estimates for association of OORP implementation with outcomes, using generalized linear models

Half-year	60-day MOUD initiation	60-day psychosocial treatment initiation	180-day overdose	180-day all-cause acute care utilization
≤-6	0.777 (0.424, 1.423)	0.739 (0.457, 1.197)	0.980 (0.746, 1.286)	1.179 (1.028, 1.353)
-5	0.868 (0.531, 1.419)	0.957 (0.630, 1.454)	0.933 (0.706, 1.233)	1.013 (0.850, 1.208)
-4	0.731 (0.417, 1.283)	0.892 (0.600, 1.325)	0.948 (0.707, 1.272)	1.164 (1.000, 1.356)
-3	0.855 (0.478, 1.530)	0.891 (0.567, 1.399)	0.841 (0.635, 1.113)	0.976 (0.850, 1.120)
-2	1.103 (0.696, 1.747)	0.694 (0.450, 1.072)	1.015 (0.789, 1.305)	1.163 (1.025, 1.319)
-1	1.112 (0.656, 1.887)	0.802 (0.537, 1.198)	0.931 (0.726, 1.194)	1.018 (0.914, 1.135)
0	REF	REF	REF	REF
1	1.476 (1.016, 2.145)	0.849 (0.592, 1.218)	0.837 (0.660, 1.061)	1.054 (0.944, 1.178)
2	1.104 (0.733, 1.664)	0.810 (0.566, 1.158)	1.023 (0.825, 1.270)	1.092 (0.966, 1.234)
3	1.209 (0.768, 1.905)	0.691 (0.486, 0.983)	0.821 (0.644, 1.047)	1.064 (0.933, 1.214)
4	0.982 (0.651, 1.480)	0.631 (0.397, 1.003)	0.727 (0.562, 0.941)	1.025 (0.906, 1.159)
5	1.377 (0.901, 2.106)	0.688 (0.407, 1.163)	0.677 (0.504, 0.910)	0.975 (0.855, 1.112)
≥6	1.556 (0.847, 2.859)	0.875 (0.499, 1.534)	0.784 (0.525, 1.170)	1.088 (0.926, 1.277)

Half-years are relative to OORP implementation, which is represented as half-year 0. MOUD and psychosocial treatment are modeled using binomial regression and treatment effects are odds ratios; overdose and acute care utilization are modeled using quasi-Poisson regression and treatment effects are incidence rate ratios. Estimates represent the difference in outcomes relative to the half-year of OORP implementation (time = 0) in patients treated in intervention vs. comparison group hospitals. Models control for variables in Table 1 and include hospital and time fixed effects. 95% confidence intervals in parentheses were calculated with standard errors clustered on hospital and patient zip code.

© 2024 Treitler P et al. *JAMA Network Open*.

eReferences.

1. Mee-Lee DE. *The ASAM Criteria: Treatment Criteria for Addictive, Substance-Related, and Co-Occurring Conditions*. 3rd ed. American Society of Addiction Medicine; 2013.

2. Centers for Medicare & Medicaid Services. Chronic Conditions Data Warehouse. Accessed November 4, 2022. https://www2.ccwdata.org/condition-categories-chronic

3. Singh GK. Area Deprivation and Widening Inequalities in US Mortality, 1969–1998. *American Journal of Public Health*. 2003;93(7):1137-1143. doi:10.2105/AJPH.93.7.1137

4. US Census Bureau. American Community Survey 5-Year Data (2009-2020). Census.gov. Published March 17, 2022. Accessed November 4, 2022. https://www.census.gov/data/developers/data-sets/acs-5year.html

5. Substance Abuse and Mental Health Services Administration. National Directory of Drug and Alcohol Abuse Treatment Facilities - 2020 | CBHSQ Data. Published April 14, 2020. Accessed November 4, 2022. https://www.samhsa.gov/data/report/national-directory-drug-and-alcohol-abuse-treatment-facilities-2020

6. U.S. Centers for Medicare & Medicaid Services. NPPES NPI Registry. Accessed May 31, 2022. https://npiregistry.cms.hhs.gov/

7. Deeks J, Higgins J, Altman D. Chapter 10: Analysing data and undertaking meta-analyses. In: Higgins J, Thomas J, Chandler J, et al., eds. *Cochrane Handbook for Systematic Reviews of Interventions, Version 6.3*. Cochrane; 2022. https://www.training.cochrane.org/handbook

8. Baker AC, Larcker DF, Wang CCY. How much should we trust staggered difference-in-differences estimates? *Journal of Financial Economics*. 2022;144(2):370-395. doi:10.1016/j.jfineco.2022.01.004

9. Cengiz D, Dube A, Lindner A, Zipperer B. The Effect of Minimum Wages on Low-Wage Jobs. *The Quarterly Journal of Economics*. 2019;134(3):1405-1454. doi:10.1093/qje/qjz014

10. Goodman-Bacon A. Difference-in-differences with variation in treatment timing. *Journal of Econometrics*. 2021;225(2):254-277. doi:10.1016/j.jeconom.2021.03.014

© 2024 Treitler P et al. *JAMA Network Open*.
