# Supplementary material for: Emergency Department Peer Support Program and Patient Outcomes After Opioid Overdose
Source: JAMA Netw Open. 2024 Mar 25;7(3):e243614. doi: 10.1001/jamanetworkopen.2024.3614 (PMC10964115; doi:10.1001/jamanetworkopen.2024.3614)
Supplement: Supplement 2. — Data Sharing Statement [file jamanetwopen-e243614-s002.pdf]

Data Sharing Statement

Treitler. Emergency Department Peer Support Program and Patient Outcomes After Opioid Overdose. *JAMA Netw Open*. Published March 25, 2024.
doi:10.1001/jamanetworkopen.2024.3614

Data
Data available: No

Additional Information
Explanation for why data not available: The data that support the findings of this study are available from the New Jersey Department of Human Services. Restrictions apply to the availability of these data, which were used under a data use agreement for this study.
